# A Qualitative Study on TPB‐Based Factors for Breastfeeding Cessation in Women With Gestational Diabetes Mellitus in the Early Postpartum Period

**DOI:** 10.1155/jp/5369691

**Published:** 2026-04-10

**Authors:** Xin Jiang, Hui Jiang, Rong Huang

**Affiliations:** ^1^ Nursing Department of Shanghai First Maternity and Infant hospital, Tongji University School of Medicine, Shanghai, China, tongji.edu.cn

**Keywords:** breastfeeding cessation, early postpartum period, gestational diabetes mellitus (GDM), postpartum women, qualitative study

## Abstract

**Objective:**

This study is aimed at exploring the core factors contributing to breastfeeding cessation in women with gestational diabetes mellitus (GDM) during the early postpartum period, so as to provide evidence‐based references for formulating targeted interventions to improve breastfeeding persistence in this population.

**Methods:**

A phenomenological qualitative research design was adopted. Semistructured in‐depth interviews were conducted with 15 GDM women who had ceased breastfeeding within 1 week after delivery. The interview data were analyzed using thematic extraction and identity to extract key themes.

**Results:**

Five core themes related to breastfeeding cessation in GDM women were identified: (1) negative evaluations shaped by inadequate and biased perceptions; (2) breastfeeding cessation driven by social pressure and conflicting influences; (3) low self‐efficacy caused by practical and physical barriers; (4) interactive effects of attitudes, norms, and perceived control; and (5) targeted support to strengthen attitudes, norms, and perceived control.

**Conclusion:**

The cessation of breastfeeding in GDM women during the early postpartum period is a result of the interaction of knowledge gaps, psychological stress, practical barriers, and inadequate support. Healthcare providers should develop individualized, GDM‐focused breastfeeding support strategies, including targeted knowledge education, timely psychological intervention, standardized clinical guidance, and family‐involved support models to reduce the rate of early breastfeeding cessation and promote long‐term breastfeeding in GDM women.

## 1. Introduction

Gestational diabetes mellitus (GDM), defined as the first onset or recognition of glucose intolerance during pregnancy, has become a major public health concern globally. The prevalence of GDM worldwide ranges from 2.3% to 17.5% [1], and in China, it has exceeded 15% due to improved living standards, the implementation of the “universal three‐child” policy, and the increasing number of advanced‐age, overweight, or obese pregnant women [2]. GDM not only increases the risk of adverse pregnancy outcomes, such as macrosomia and neonatal hypoglycemia, but also elevates the long‐term risk of metabolic diseases, including Type 2 diabetes, obesity, and hypertension in both mothers and their offspring [3–5].

Breastfeeding, recognized as the optimal infant feeding method by the World Health Organization (WHO), offers significant health benefits for both GDM mothers and their infants. For mothers, it reduces the risk of developing Type 2 diabetes later in life [5]; For infants, it enhances immune function, promotes long‐term metabolic health, and reduces the risk of obesity and diabetes in adulthood. However, numerous studies have consistently shown that compared with women with normal blood glucose during pregnancy, GDM women tend to cease breastfeeding earlier, with a significantly lower rate of exclusive breastfeeding in the early postpartum period [6–8]. The first week after delivery is a critical window for determining whether women can maintain exclusive breastfeeding, and the status of early breastfeeding directly affects the duration of long‐term breastfeeding [9].

Despite the well‐documented importance of breastfeeding for GDM mothers and infants, the factors leading to breastfeeding cessation in this specific population remain not fully understood. Most existing studies have focused on the general experiences of breastfeeding in GDM women, whereas few have specifically explored the mechanisms and core factors underlying breastfeeding cessation. To address this gap, the present qualitative study will draw on the theory of planned behavior (TPB) as a theoretical framework to guide the investigation.

The TPB posits that human behavior is primarily shaped by three core factors: attitudes toward the behavior, subjective norms, and perceived behavioral control [10]. Attitudes refer to the individual′s positive or negative evaluation of performing the behavior; subjective norms reflect the perceived social pressure from significant others to engage in or refrain from the behavior; and perceived behavioral control involves the individual′s belief in their ability to successfully perform the behavior, which is closely linked to self‐efficacy. Together, these factors influence an individual′s behavioral intention, which in turn predicts actual behavior.

Although existing literature has examined breastfeeding experiences and challenges among women with GDM across the postpartum continuum [11], and the TPB has been applied to predict breastfeeding initiation and duration in various populations [12], a critical gap remains regarding the mechanisms underlying very early breastfeeding cessation, specifically within the first 72 h to 1 week postpartum in this high‐risk population.

Current evidence suggests that women with GDM may experience delayed Lactogenesis II [13], and that breastfeeding cessation occurs most steeply during the first 2 weeks after birth. However, existing qualitative studies have primarily captured retrospective accounts of breastfeeding interruption without distinguishing between acute cessation decisions made in the immediate postpartum period versus gradual weaning processes [14]. Furthermore, although TPB constructs have demonstrated predictive utility for breastfeeding behavior among general parturient women, the dynamic interplay between these cognitive factors and the unique physiological and contextual stressors of early GDM management remains theoretically underdeveloped.

Specifically, there is limited understanding of how intention‐behavior discordance manifests in the immediate postpartum period for women with GDM;particularly how rapidly shifting perceptions of milk insufficiency and blood glucose monitoring requirements interact with prenatal breastfeeding intentions to precipitate early cessation decisions. The volatile decision‐making environment of the early postpartum period may not be adequately captured by quantitative research, during this time, acute physiological barriers and contextual pressures may override prenatal intentions before breastfeeding is fully established.

Therefore, this qualitative study adopts a phenomenological approach to conduct in‐depth interviews with women with GDM who discontinued breastfeeding within the first week postpartum. Under the guidance of the TPB, this study can more deeply reveal the essential issues and underlying mechanisms behind the decision to discontinue breastfeeding among women with GDM in the immediate postpartum period. By focusing on the three core dimensions of attitude, subjective norm and perceived behavioral control; this study is aimed at exploring the key essential factors leading to early breastfeeding cessation, so as to provide a theoretical basis for developing targeted and time‐sensitive interventions, which can precisely address the critical window during which the breastfeeding trajectory of women with GDM is most vulnerable to disruption.

## 2. Materials and Methods

### 2.1. Study Design

This study employed a qualitative thematic approach deductively guided by the TPB. Semi‐structured in‐depth interviews were conducted to explore participants′ perceptions, attitudes, and behavioral intentions around the early breastfeeding cessation. The researches organized data analysis around the core constructs of the TPB including attitude, subjective norm, perceived behavioral control and intention. Thematic extraction and analysis was used to identify, code and report key patterns and themes derived from the interview data with the theoretical framework providing a structured deductive approach to data interpretation. This design allowed for a systematic exploration of participants′ experiences within a clear theoretical structure.

### 2.2. Study Participants

Purposive sampling was used to recruit participants from a tertiary maternal and infant hospital in Shanghai, China, between Jan and Mar 2025. The inclusion criteria were as follows: (1) diagnosed with GDM according to the diagnostic criteria recommended by the International Association of Diabetes and Pregnancy Study Groups (IADPSG) [15]; (2) singleton pregnancy; (3) no absolute contraindications to breastfeeding, such as active tuberculosis and HIV infection; (4) ceased breastfeeding or switched to formula feeding within 1 week after delivery; (5) no communication barriers, able to express personal experiences clearly, and voluntarily consented to participate in the study. The exclusion criteria were as follows: (1) postpartum mother–infant separation due to maternal or infant health issues and (2) withdrew from the interview midway or failed to complete the entire interview process. A total of 15 participants were included, numbered as N1–N15. The general demographic characteristics of the participants are shown in Table 1.

**Table 1:** General demographic characteristics of participants.

Number	Age (years)	Educational background	Parity	Mode of delivery	Neonatal condition	Duration of breastfeeding (days)
N1	31	College	G3P2	Forceps delivery	Normal weight	3
N2	32	Undergraduate	G1P1	Vaginal delivery	Macrosomia	2
N3	31	Postgraduate	G5P1	Cesarean section	Normal weight	4
N4	29	Undergraduate	G4P3	Cesarean section	Neonatal hypoglycemia	1
N5	33	Senior high school	G4P1	Vaginal delivery	Neonatal jaundice	3
N6	41	College	G2P1	Cesarean section	Neonatal hypoglycemia	2
N7	27	Undergraduate	G1P1	Cesarean section	Macrosomia, hypoglycemia	1
N8	38	Undergraduate	G2P2	Cesarean section	Neonatal hypoglycemia	2
N9	31	College	G1P1	Cesarean section	Macrosomia, hypoglycemia	3
N10	31	College	G4P2	Cesarean section	Neonatal hypoglycemia	2
N11	32	Undergraduate	G2P1	Vaginal delivery	Macrosomia, jaundice	4
N12	31	Undergraduate	G2P2	Vaginal delivery	Neonatal jaundice	3
N13	27	Undergraduate	G2P1	Vaginal delivery	Neonatal hypoglycemia	1
N14	34	Senior high school	G4P2	Cesarean section	Neonatal jaundice	4
N15	30	Undergraduate	G1P1	Vaginal delivery	Macrosomia	2

### 2.3. Data Collection

Semistructured in‐depth interviews were conducted to collect data. Before each interview, the researcher explained the purpose, methods, confidentiality measures, and right to withdraw of the study to the participants. After obtaining written informed consent, a general demographic questionnaire was completed.

Interviews were conducted in a quiet and private environment to ensure the participants′ comfort and willingness to share. For in‐hospital participants, interviews were held in the obstetric conference room after routine treatment and when the infant was asleep. For participants who had been discharged, interviews were conducted via the telephone. Data were collected through semistructured interviews using both face‐to‐face and telephone approaches. Face‐to‐face interviews were conducted in a quiet and private setting to establish rapport and enable in‐depth communication, whereas telephone interviews were adopted for participants unable to attend in‐person meetings, as this method was more convenient and acceptable for GDM mothers in the early postpartum period. No notable differences in interview depth, emotional expression, or the emergence of main themes were observed between the two modalities, and the duration of telephone interviews was comparable with that of in‐person interviews. The same interview outline, probing strategies, and researcher were applied throughout all interviews to ensure consistency in data collection procedures and data quality.

Guided by the TPB, the interview outline was developed based on a comprehensive literature review and the research purpose covering the following core topics aligned with the theory′s core constructs:

1. Attitudes toward breastfeeding: Your evaluation of breastfeeding, such as perceived benefits, drawbacks, or value before and after delivery, particularly regarding its impact on your GDM management including blood glucose control and postpartum recovery and your infant′s health. Did these evaluations change over time, and if so, how?
2. Subjective norms related to breastfeeding cessation: The opinions or pressures from significant others like partners, parents, in‐laws, healthcare providers regarding whether to continue or stop breastfeeding. How did these social influences shape your decision to cease breastfeeding?
3. Perceived behavioral control over breastfeeding: The difficulties or challenges you encountered during breastfeeding, such as physical discomfort, milk supply issues, and time constraints balancing breastfeeding with GDM care, and your perceived ability to overcome them. To what extent did you feel capable of maintaining breastfeeding despite these challenges?
4. Behavioral intention and decision‐making process: The sequence of thoughts and actions leading to your decision to stop breastfeeding; including how your attitudes, social influences, and perceived control interacted to form your intention to cease breastfeeding?
5. Expectations for enhancing breastfeeding persistence: What types of support including educational guidance, practical assistance, and emotional encouragement do you believe would strengthen your perceived control over breastfeeding or improve your attitudes toward it, helping GDM mothers continue breastfeeding longer?

During the interviews, we used open‐ended questions and timely follow‐ups to explore participants′ true experiences and feelings; (“Can you tell me more about that?” “How did you feel at that time?”) Each participant was interviewed 1 to 2 times, lasting 30 to 40 min. All interviews were audio‐recorded, and detailed field notes were taken to record the participants′ facial expressions, tone of voice, and body language.

Data saturation was assessed during data collection and analysis. Recruitment was continued until no new themes or concepts related to the research questions emerged; in this study, data saturation was achieved after interviewing 15 participants, with no additional new themes identified in the final three interviews [16].

### 2.4. Data Analysis

Within 24 h after each interview, the audio recordings were transcribed verbatim into text; and the field notes were integrated to form a complete interview manuscript. The data were analyzed using Colaizzi′s 7‐step method [17], which includes the following steps: (1) read all interview transcripts repeatedly to gain a comprehensive understanding of the data; (2) extract statements with important meanings related to breastfeeding cessation; (3) code and categorize the repeated meaningful statements; (4) integrate the coded data to form initial themes or theme clusters; (5) describe the phenomenon in detail without omitting important information; (6) refine and elevate the initial themes to form core theme concepts; and (7) return to the participants to verify the consistency between the analysis results and their true experiences, ensuring the credibility of the data.

Data analysis was performed using the above analysis method. After transcription, two researchers independently coded the transcripts line by line. Initial codes were generated, discussed, and categorized into subthemes and main themes through repeated comparison and refinement. Any discrepancies were resolved through group discussion until a consensus was reached.

NVivo 12 software was used to assist data management and coding. An audit trail was established throughout the research process, including raw data, coding records, and decision notes; to ensure the transparency and traceability of the analysis. To enhance credibility, peer debriefing was adopted to verify the coding process and thematic structure.

### 2.5. Rigor and Ethical Considerations

To ensure the rigor of the study, the following measures were implemented: (1) the researcher received professional training in qualitative research methods and breastfeeding knowledge, and the interview outline was reviewed and revised by one breastfeeding expert and two qualitative research experts; (2) before the formal investigation, the researcher fully recognized the potential influence of professional background and preconceptions on data collection and analysis. During the study, reflective notes were written after each interview to record personal thoughts, emotional responses, and biases that might affect the research process. (3) consistent interview guidelines and coding strategies were adopted, and regular discussions with experienced qualitative researchers were conducted to assist reflection, reduce subjective bias, and ensure the rigor and credibility of the study; (4) purposive sampling was used to include participants with different ages, educational backgrounds, parity, and modes of delivery to ensure sample representativeness; (5) member checking was conducted by returning the analysis results to the participants for verification; and (6) peer review was performed by qualitative research experts to evaluate the consistency between the original data and the research results.

Prior to the study, ethical approval was obtained from the Ethics Committee of the participating hospital (Approval No: KS24455). Ethical principles were strictly followed throughout the study.(1) informed consent: Participants were fully informed of the study details and could withdraw at any time without negative consequences; (2) confidentiality: All participants were assigned numbers instead of real names, and audio recordings, transcripts, and questionnaires were stored in a locked cabinet and encrypted electronically to protect participants′ privacy; (3) respect for autonomy: Participants′ views and feelings were respected, and no suggestive or leading language was used during interviews.

## 3. Results

### 3.1. Demographic Characteristics of Participants

A total of 15 GDM women who ceased breastfeeding in the early postpartum period were included in this study. In terms of age, the participants ranged from 27 to 41 years old, with an average age of (32.13 ± 3.92) years. There were two participants with a senior high school education, four participants with a college education, and eight participants with an undergraduate education. Nine primiparous and six multiparous women were included. Six participants had vaginal deliveries, and eight participants underwent cesarean sections; In addition, there was also one participant who had a forceps delivery. Regarding the duration of breastfeeding, the participants stopped breastfeeding within 1–4 days after delivery. The shortest duration was 1 day, and the longest was 4 days. All participants ceased breastfeeding in the early postpartum period.

### 3.2. Main Themes

Through in‐depth analysis of the interview data, and guided by the TPB, four core themes contributing to breastfeeding cessation in GDM women were extracted, which correspond to the key dimensions of the theory.

#### 3.2.1. Attitudes Toward Breastfeeding: Inadequate and Inaccurate Perceptions Shaping Negative Evaluations

Participants′ attitudes toward breastfeeding were largely shaped by their limited understanding of its specific value for GDM mothers and infants; leading to weak commitment to persistence when challenges arose.

##### 3.2.1.1. Undervaluation of Breastfeeding′s Unique Benefits for GDM Dyads.

Participants generally recognized basic advantages of breast milk, such as more nutritious than formula, but lacked awareness of its long‐term health impacts tailored to their condition. Specifically, they were unaware that breastfeeding reduces GDM mothers′ risk of type 2 diabetes and mitigates infants’ metabolic disease risks; knowledge gaps that weakened their motivation to persist.

During pregnancy, I learned all about blood glucose control but nothing about breastfeeding′s role in my future health. If I′d known it lowers diabetes risk, I might have tried harder. (N4)

I thought formula was an acceptable substitute. No one emphasized that breastfeeding is especially critical for babies of GDM mothers, so quitting felt easy. (N9)

##### 3.2.1.2. Shifting Evaluations Due to Practical Challenges.

Initial positive attitudes (“breast milk is best”) deteriorated as participants encountered difficulties, with many revising their perceptions to view breastfeeding as “ineffective” or “harmful” for their infants. For example, mothers of hypoglycemic infants began to doubt breastfeeding′s ability to stabilize blood glucose, whereas those with macrosomic babies questioned its sufficiency.

When my baby′s hypoglycemia persisted, I started thinking breast milk wasn′t keeping him safe. Formula felt more “reliable”. (N13)

My macrosomic baby cried constantly after breastfeeding. I decided breast milk just wasn′t enough, even though I wanted to keep trying. (N15)

#### 3.2.2. Subjective Norms: Social Pressures and Conflicting Influences Driving Cessation

Participants′ decisions were heavily influenced by the opinions of significant others, including healthcare providers, family members, and spouses, whose mixed messages or pressure to switch to formula undermined breastfeeding persistence.

##### 3.2.2.1. Conflicting Guidance From Healthcare Providers.

Inconsistent advice from obstetric and pediatric staff created confusion; with participants often prioritizing recommendations that favored formula. For instance, nurses advocating exclusive breastfeeding clashed with pediatricians emphasizing “controlled feeding” for macrosomic or hypoglycemic infants.

The nurse said “breastfeed on demand,” but the pediatrician insisted on scheduled formula feeds for my baby′s hypoglycemia. I trusted the doctor more and stopped breastfeeding. (N6)

One doctor said breastfeeding helps with my blood glucose; another said it might stress me out. I didn′t know who to believe, so I gave up. (N8)

##### 3.2.2.2. Traditional Family Beliefs Undermining Breastfeeding.

Elderly relatives (mothers and mothers‐in‐law) frequently challenged breastfeeding; citing myths like “colostrum is insufficient” or “formula ensures weight gain,” creating emotional pressure to quit.

My mother‐in‐law kept saying, “Your milk isn′t nutritious enough—look how skinny the baby is.” Every time he cried; she pushed formula. Eventually, I gave in. (N7)

My mom insisted that GDM meant my milk was “unhealthy.” Her constant doubt made me question myself too. (N5)

##### 3.2.2.3. Limited Spousal Support.

Although spouses provided practical care, such as cooking or laundry, they rarely engaged with breastfeeding education or emotional support, leaving participants feeling unsupported. Their lack of involvement reinforced the perception that breastfeeding was a “solitary burden.”

My husband never learned about breastfeeding. When I struggled, he just said, ‘Do what′s easiest.” That made me feel that quitting was like the only option. (N10)

#### 3.2.3. Perceived Behavioral Control: Low Self‐Efficacy due to Practical and Physical Barriers

Participants′ sense of control over breastfeeding was eroded by challenges they felt unable to overcome including lactation issues, infant health concerns, and the demands of GDM management.

##### 3.2.3.1. Lactation Difficulties Reducing Self‐Efficacy.

Delayed milk onset, perceived insufficient supply, and difficulty managing breastfeeding for high‐need infants (e.g., macrosomic and hypoglycemic) led participants to feel incapable of successful breastfeeding.

I couldn′t get my milk to come in, and my baby cried nonstop. I felt completely powerless—like I was failing him. (N11)

My hypoglycemic baby needed frequent feeds, but I had no idea how to balance that with my own blood glucose checks. It was too overwhelming. (N13)

##### 3.2.3.2. Physical Discomfort and Recovery Challenges.

Cesarean section pain, nipple soreness, and fatigue from nighttime feeds compounded feelings of inadequacy with many believing they lacked the physical capacity to persist.

After my C‐section, even holding the baby to breastfeed hurt; I didn′t think I could keep going, so I switched to formula. (N3)

##### 3.2.3.3. Balancing GDM Care With Breastfeeding.

The demands of blood glucose monitoring, dietary restrictions, and medication management left participants feeling they lacked the bandwidth to prioritize breastfeeding.

I was so focused on checking my blood sugar 6 times a day that breastfeeding felt like one chore too many; I couldn′t handle both. (N6)

#### 3.2.4. Behavioral Intention and Decision‐Making: Interaction of Attitudes, Norms, and Control

The decision to stop breastfeeding emerged from the interplay of weak attitudes (undervaluing benefits), negative subjective norms (pressure to quit) and low perceived control (inability to overcome challenges). This interaction created a clear intention to abandon breastfeeding.

For example, N9 initially wanted to breastfeed but shifted her intention after: (1) learning little about its benefits for GDM (weak attitude), (2) her mother‐in‐law′s constant criticism (negative norm), and (3) struggling to feed her macrosomic baby (low control). “It all added up,” she explained. “I didn′t see the point in fighting anymore.”

Similarly, N4′s intention to quit solidified when her doctor′s vague advice (norm) clashed with her fear of insufficient milk (control) and her lack of knowledge about breastfeeding′s role in her diabetes risk (attitude). “Everything pushed me toward formula,” she noted.

#### 3.2.5. Expectations for Enhancing Persistence: Targeted Support to Strengthen Attitudes, Norms, and Control

Participants identified specific supports needed to improve breastfeeding outcomes, aligned with TPB dimensions.

##### 3.2.5.1. Educational Interventions to Strengthen Attitudes.

They requested tailored education on breastfeeding′s long‐term benefits for GDM mothers (e.g., diabetes prevention) and infants (e.g., metabolic health), delivered prenatally and postnatally.

I wish someone had explicitly told me breastfeeding could lower my diabetes risk. That would have made me fight harder. (N4)

##### 3.2.5.2. Consistent Guidance to Improve Subjective Norms.

Participants sought unified advice from healthcare teams (reducing conflicting norms) and family education to counter traditional myths.

If doctors and nurses all said the same thing, and my mother‐in‐law understood why breastfeeding matters, I might have persisted. (N7)

##### 3.2.5.3. Practical Support to Boost Perceived Control.

They needed hands‐on help with lactation techniques (e.g., stimulating milk supply for macrosomic infants), pain management (e.g., nipple care), and tools to balance GDM care with breastfeeding (e.g., synchronized feeding/glucose check schedules).

A lactation consultant who knew about GDM could have taught me how to handle my baby′s hypoglycemia while breastfeeding. I felt so alone figuring it out. (N13)

##### 3.2.5.4. Peer and Spousal Engagement.

Participants desired support groups for GDM mothers to share experiences (strengthening control through shared solutions) and spousal education to foster active involvement (improving norms).

Connecting with other GDM moms who breastfed would have made me feel less alone. And if my husband knew how to help, I wouldn′t have quit. (N15)

## 4. Discussion

Our study guided by the TPB systematically explores the mechanisms underlying early breastfeeding cessation among GDM women and highlighting the interplay of attitudes, subjective norms, and perceived behavioral control in shaping breastfeeding intentions. The findings not only validate the applicability of TPB in explaining breastfeeding behaviors within this specific population; but also reveal unique contextual factors that require targeted intervention.

### 4.1. Attitudes: Knowledge Gaps Undermine Commitment

The study identifies a critical link between inadequate knowledge of breastfeeding′s GDM‐specific benefits and weak attitudes, which directly reduce mothers′ resolve to persist. Participants′ general awareness of breast milk′s nutritional advantages (e.g., “more nutritious than formula”) contrasts sharply with their ignorance of its long‐term protective effects, such as reducing maternal Type 2 diabetes risk and mitigating infant metabolic vulnerabilities. This aligns with cross‐sectional studies showing that mothers who recognize breastfeeding as “essential” for their health are 2.3 times more likely to sustain breastfeeding beyond 6 months [12, 13].

Notably, attitudes shifted negatively when challenges arose; as mothers reinterpreted breastfeeding as “ineffective” for infants with complications like hypoglycemia or macrosomia. This plasticity in attitudes underscores the need for dynamic, context‐responsive education. For example, prenatal counseling should emphasize breastfeeding′s role in stabilizing neonatal blood glucose [18, 19], whereas postnatal support should address misconceptions, such as “breast milk is insufficient for macrosomic infants” before they solidify into negative beliefs [20].

### 4.2. Subjective Norms: Conflicting Influences Create Decision Paralysis

Subjective norms emerged as a double‐edged sword, with both healthcare providers and family members exerting significant, often contradictory, influence. Conflicting guidance from clinicians, such as obstetric nurses advocating exclusive breastfeeding versus pediatricians prioritizing formula for “controlled feeding”—undermines maternal confidence, as observed in similar studies of high‐risk pregnancies [21]. This inconsistency reflects a systemic gap in interdisciplinary care: GDM management and lactation support remain siloed, with few protocols integrating both [22].

Family pressure, particularly from elderly relatives, further complicates decision‐making. Traditional myths (e.g., “colostrum is weak” or “GDM milk is unhealthy”) persist due to limited health literacy and cultural norms that prioritize infant weight gain over breastfeeding [23]. Spousal disengagement exacerbates this, as partners′ lack of involvement reinforces the perception that breastfeeding is a “woman′s burden”—a finding consistent with qualitative work showing that spousal support is a stronger predictor of breastfeeding duration than maternal age or education [24].

### 4.3. Perceived Behavioral Control: Cumulative Barriers Erode Self‐Efficacy

Perceived control was eroded by interconnected challenges: lactation difficulties, physical discomfort, and the cognitive load of GDM management. For GDM mothers, these barriers are amplified by the need to synchronize breastfeeding with blood glucose monitoring, dietary restrictions, and infant health crises (e.g., hypoglycemia episodes). This aligns with TPB′s assertion that perceived control mediates the relationship between intention and behavior [25, 26]; when mothers feel incapable of overcoming obstacles, even strong intentions falter.

Lactation issues—delayed onset, perceived insufficiency, and struggles with high‐need infants—were particularly damaging to self‐efficacy. Unlike non‐GDM mothers, who often attribute low milk supply to “temporary adjustment,” GDM mothers linked it to their condition (e.g., “my GDM makes my milk inadequate”), creating a self‐reinforcing cycle of doubt [27]. Physical barriers, such as cesarean pain or nipple soreness, compounded this, with 67% of participants reporting that pain “directly made me want to quit”—a rate 1.8 times higher than in non‐GDM cohorts [28].

### 4.4. Interaction of TPB Constructs: A Vicious Cycle of Cessation

The decision to stop breastfeeding arose not from isolated factors but from the synergistic interplay of weak attitudes, negative norms, and low control. For instance, N9′s trajectory—undervaluing breastfeeding′s benefits (attitude), facing maternal criticism (norm), and struggling with a macrosomic infant (control)—illustrates how each construct amplifies the others, creating an “exit threshold” that is crossed when the perceived cost of persistence outweighs the benefits. This aligns with TPB′s predictive model, where intention is a function of attitudes (*β* = 0.32), norms (*β* = 0.28), and control (*β* = 0.35) [29].

### 4.5. Implications for Intervention Design

The findings support a TPB‐informed intervention framework encompassing three core components: attitude‐strengthening through prenatal workshops that utilize decision aids (e.g., infographics illustrating the link between breastfeeding and reduced diabetes risk) alongside postnatal “myth‐busting” sessions addressing complications such as neonatal hypoglycemia; norm‐alignment via interdisciplinary training for clinicians to deliver unified guidance (e.g., “breastfeed every 2 hours to stabilize blood glucose”) and family education programs targeting elderly relatives and spouses; and control‐enhancing measures involving the deployment of GDM‐specialized lactation consultants to teach techniques like breast compression for macrosomic infants [30, 31] and provide tools such as synchronized feeding/glucose check schedules to reduce cognitive load. Based on similar TPB‐based programs implemented for low‐income mothers [30, 32], such interventions could potentially increase exclusive breastfeeding rates among GDM women.

### 4.6. Limitations

Several limitations should be acknowledged. First, participants were recruited from a single tertiary hospital. This may restrict the generalizability of the findings; Second, given the specific characteristics of GDM mothers and the single‐center design, caution should be exercised when transferring the findings to other populations or clinical settings. These limitations should be taken into account when interpreting the results of this study.

## 5. Conclusion

In summary, this study demonstrates that early breastfeeding cessation among GDM women is a predictable outcome of modifiable factors including weak attitudes, conflicting norms, and eroded control rather than inevitable. By addressing these factors through TPB‐aligned strategies, healthcare systems can empower GDM mothers to overcome barriers, and realize breastfeeding′s full benefits for both themselves and their infants.

## Author Contributions

Xin Jiang designed the study, conducted the analysis, and drafted the initial manuscript. She also provided substantial contributions to the design of the study and interpretation of the data. Hui Jiang and Rong Huang are responsible for the postrevision of the manuscript.

## Funding

This study was supported by the Research Project on Normative Systems for Health Governance and Rule of Law; Shanghai Municipal Health Commission and Nursing Research Project of the Chinese Medical Association, CMAPH‐NRP2022008.

## Disclosure

All authors reviewed and revised the manuscript and approved the final manuscript as submitted.

## Ethics Statement

All aspects of the study were approved by the Ethics Review Board of the Shanghai First Maternity and Infant Hospital (No. KS24455) and written informed consent was obtained from caregivers before the interview.

## Consent

The authors have nothing to report.

## Conflicts of Interest

The authors declare no conflicts of interest.

## Data Availability

The data that support the findings of this study are available on request from the corresponding author. The data are not publicly available due to privacy or ethical restrictions.
